# The use of the sodium fluorescein and YELLOW 560 nm filter for the resection of pediatric posterior fossa lesions

**DOI:** 10.1007/s00381-022-05798-9

**Published:** 2022-12-17

**Authors:** Christian Ott, Martin Proescholdt, Monika Friedrich, Julius Hoehne, Katharina Rosengarth, Nils-Ole Schmidt, Karl-Michael Schebesch

**Affiliations:** Department of Neurosurgery, University Hospital Regensburg, Regensburg, Germany

**Keywords:** Neurosurgery, Posterior Fossa, Child, Fluorescein

## Abstract

**Purpose:**

This study aimed to verify the feasibility, safety, and benefit of using fluorescein sodium (FL) and a YELLOW 560 nm filter in posterior fossa tumors in children.

**Methods:**

All cases of pediatric posterior fossa tumors that have undergone surgery using fluorescein (2018–2022) have been included and were examined retrospectively. In those cases where resection of the tumor was planned, a blinded neuroradiologist distinguished gross total resection and subtotal resection according to the postoperative MRI findings. The surgical report and medical files were reviewed regarding the intraoperative staining grade and adverse events. The grade of fluorescent staining of the targeted lesion was assessed as described in the surgical reports. The screening was conducted for any reference to the degree of fluorescent staining: “intense,” “medium,” “slight,” and “no staining.”

**Results:**

19 cases have been included. In 14 cases, a complete resection was initially intended. In 11 of these cases, a gross total resection could be achieved (78.6%). Staining was described as intense in most cases (58.8%). Except for yellow-colored urine, no side effects obviously related to FL were found throughout the observation period.

**Conclusion:**

In combination with a specific filter, FL is a reliable, safe, and feasible tool in posterior fossa surgery in children.

## Introduction

Pediatric CNS tumors are the second-most common malignancy in childhood. They are regarded as the most common solid tumor in children and the leading cause of death from solid tumors in childhood in the USA [1].

The most common tumors in the posterior fossa during childhood are medulloblastoma (MB), atypical teratoid/rhabdoid tumor (ATRT), pilocytic astrocytoma (PA), ependymoma, and brainstem glioma [2].

For most malignant posterior fossa tumors, there is a threefold treatment strategy consisting of maximal safe resection, radiotherapy, and chemotherapy [2, 3].

But even in low-grade tumors, e.g., a cerebellar pilocytic astrocytoma, the surgical goal is a maximal safe resection, as gross total resection leads to excellent survival rates [2, 4].

With the exclusion of brainstem gliomas, the surgical goal in posterior fossa tumors in childhood is maximal safe resection.

The intraoperative optical distinction between tumor and brain tissue can be challenging, especially at the tumor’s margins. This can result in incomplete resection, although complete macroscopic surgical resection (gross total resection (GTR)) would often be feasible.

Several technical adjuncts based on fluorophores have been examined with the purpose of significantly and safely increasing the extent of resection, including fluorescein [5]. The sodium salt of fluorescein is a water-soluble organic dye that accumulates in areas with a disrupted blood–brain barrier (BBB) [6].

The mode of action of FL is similar to radiocontrast agents (e.g., gadolinium) for magnetic resonance imaging. The intravenous administration is known for its low rates of adverse reactions and its high tolerability [5].

FL has been used in retinal angiography since 1961 [7], and the first neuro-oncological reports of the use of FL date back to the year 1947 [8].

The first experiences using FL were made without any specific filter using high dosages of FL.

Through the introduction of filters into the microscope, the dosage of FL could be reduced. These filters were adapted to the absorption and emission wavelength peaks of FL to maximize the visualization of tumor tissue [5].

Dedicated FL-specific microscope filters impelled a renaissance of FL-guided surgery in brain tumors. (For example, YELLOW 560-filter on the Pentero microscope (Carl Zeiss Meditec, Germany)) [9].

The use of this fluorophore, in combination with the YELLOW560 filter, improves visualization and, thus, resection of several types of tumors of the central nervous system [10–12].

This study aimed to verify the feasibility, safety, and benefit of using FL in posterior fossa tumors in children.

## Methods

### Patients

The institutional review board approved the study (Z-2015–0478-9, Ethics Committee of the University of Regensburg).

Informed consent was obtained from all parents for the off-label use of FL.

Inclusion criteria were patients suffering from gadolinium-enhancing cerebellar neoplasm under the age of 18 years. Exclusion criteria were patients with impaired liver or renal function or a history of allergic reactions against FL.

### Pre- and postoperative clinical and neuroradiological assessment

All patients (and cases) had previously undergone a gadolinium-enhanced MRI scan. Each patient received contrast-enhanced MRI within 72 h after surgery to rule out hemorrhage and to confirm the extent of resection (EOR). A blinded neuroradiologist distinguished two categories: no residual tumor tissue = gross total resection (GTR) and residual tumor tissue = subtotal resection (STR).

### Surgical protocol

All patients were administered a weight-adjusted dose (2 mg/kg body weight) of sodiumfluorescein. If en bloc surgery as the preferred method of tumor resection was not feasible, tumors were removed in a piece-meal fashion under fluorescence visualization with a YELLOW 560 filter integrated into the microscope.

When applicable, ultrasound, neuromonitoring, and neuronavigation were used for craniotomy planning, anatomical orientation, and tumor resection.

The grade of fluorescent staining of the targeted lesion was assessed as described in the surgical reports. The screening was conducted for any reference to the degree of fluorescent staining: “intense,” “medium,” “slight,” and “no staining.”

The medical reports were evaluated for any possible adverse effect or allergic reaction to FL.

## Results

Overall, 16 patients (9 female, 7 male) with a mean age of 8.7 ± 4.1 years had undergone FL-guided surgery for resection (or partial resection) of a cerebellar tumor at our department between 2018 and 2022. All in all, 19 surgical procedures were performed, as three patients have undergone surgery twice.

In 11 cases, a gross total resection could be performed. Two patients received a planned open biopsy, whereas in 3 cases, the surgery was initially intended as a partial resection. In the remaining 3 cases, a subtotal resection was performed unintendedly.

### Extent of resection

In 3 cases, a planned partial resection (PPR) was performed, whereas 3 cases were intended as an open biopsy (OB). Of the remaining 14 cases, 11 (78.6%) received a gross total resection (GTR) and 3 (21.4%) a subtotal resection (STR) (Table 1).

**Table 1:** Parameters of the cases included in this study

Case	Pat	Age	Histopathology	EOR	Localization	Staining	Symptoms
1	1	10.7	Medulloblastoma (IV)	GTR	Left cerebellum	+ + +	Headache, vomiting, ataxia, speech disorder
2	2	7.1	Pontine glioma (IV)	OB	Pons and right cerebellum	+ + +	Headache, nausea, ataxia
3	3	2.7	Pilozytic astrozytoma (I)	GTR	Left cerebellum	+ + +	Ataxia, vomiting, diplopia
4	4	16.6	Dysplastic gangliozytoma (I)	OB	Left cerebellar peduncle, left hypothalamus, left cerebellum, pons, and vermis	+	Headache
5	5	11.9	Pilozytic astrozytoma (I)	STR	Vermis and 4th ventricle	+ + +	Headache, vertigo
6	6	6.8	Pilozytic astrozytoma (I)	GTR	Right cerebellum	+ + +	Ataxia, vomiting, impaired vision, papilledema
7	7	0.7	Atypical teratoid rhabdoid tumor (IV)	PPR	Right cerebellopontine angle	(-)	Headache, vomiting, caudal nerve palsy
8	8	14.9	Malignant germ cell tumor	GTR	Pineal region, tectum, and 3rd ventricle	(-)	Headache, vomiting, diplopia
9	9	6.5	Medulloblastoma (IV)	GTR	4th ventricle and medulla oblongata	+	Nausea, vomiting
10	10	4.3	Medulloblastoma (IV)	GTR	4th ventricle, foramen magnum, and tectum	+ + +	None
11	11	12.9	Medulloblastoma (IV)	GTR	Right cerebellopontine angle and right cerebellum	+ + +	None
12	11	13.2	Necrosis*	GTR	Foramen magnum	+ + +	None
13	12	8.7	Medulloblastoma (IV)	STR	Left cerebellum	0	Nausea, vomiting
14	12	8.7	Medulloblastoma (IV)	GTR	Left cerebellum	0	none
15	13	6.8	Pilozytic astrozytoma (I)	PPR	4th ventricle	+ + +	Vomiting
16	14	3.5	Medulloblastoma (IV)	GTR	4th ventricle	+	Headache, vomiting
17	15	9.2	Atypical ependymoma (III)	PPR	4th ventricle	+ +	Headache, papilledema
18	15	9.2	Atypical ependymoma (III)	STR	4th ventricle	+ +	None
19	16	11.9	Pilozytic astrozytoma (I)	GTR	Right cerebellum	+ + +	None

*Case* case number, *pat* patient number, *age* years, *histopathology* histopathological findings (WHO grade in brackets), *EOR* extent of resection, *GTR* gross total resection, *OB* open biopsy, *STR* subtotal resection, *PPR* planned partial resection), *localization* tumor localization, *staining* (staining grade of fluorescein during surgery (+ +  + intense, +  + medium; + slight, 0 no staining, (0) no information), and symptoms (initial symptoms of the patient)

*No vital tumor tissue was found

### Staining

**Fig. 1:**
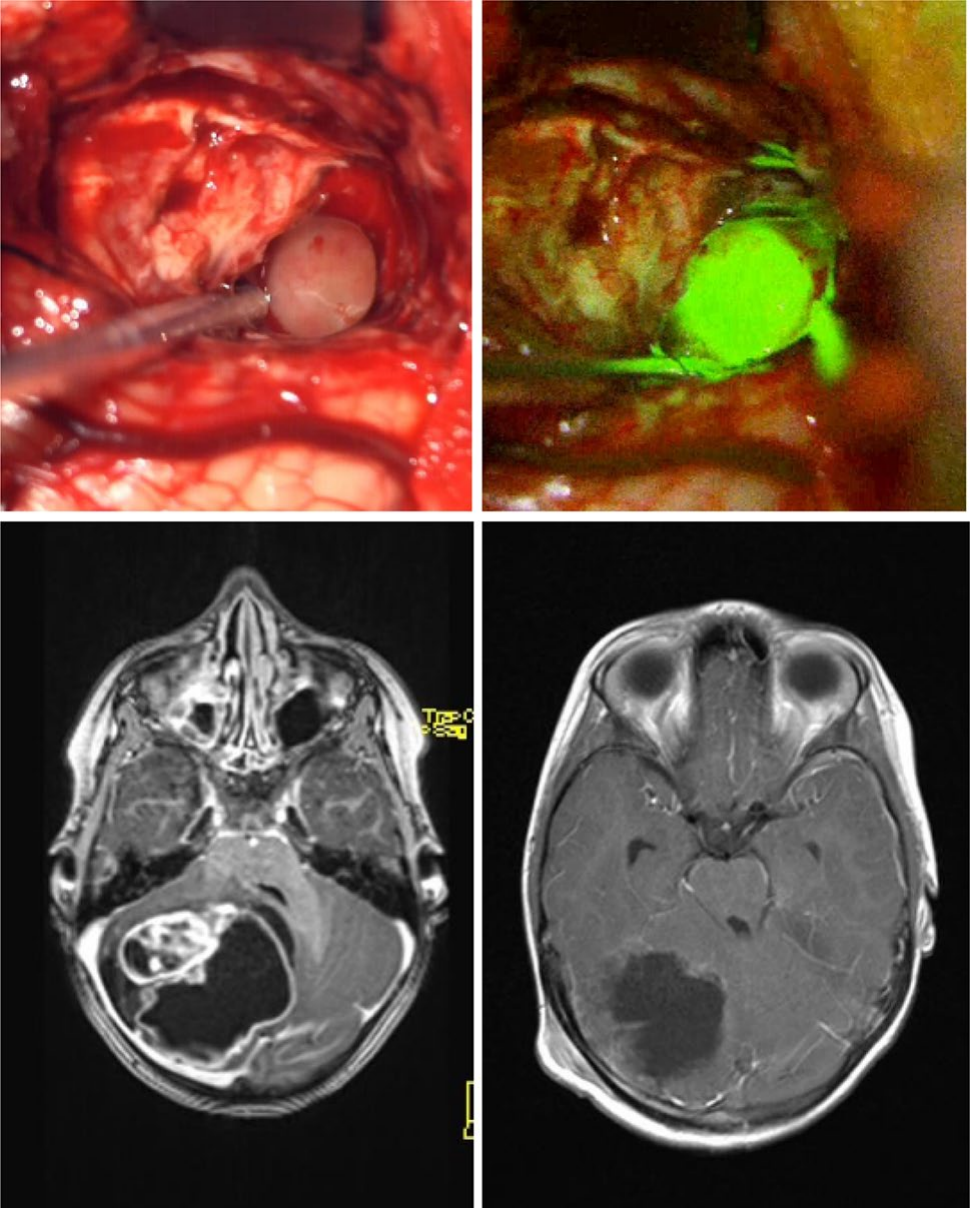
Illustration of intraoperative findings and pre-and postoperative MRI (Pat.-No.: 6). The upper images show the intraoperative images with (right) and without (left) the fluorescence as seen through the microscope. The bottom row shows the preoperative MRI (left) and postoperative MRI (right) of the same patient (T1 with gadolinium enhancement)

In two cases, no reliable information about the staining grade was found. From the remaining 17 cases, 10 (58.8%) showed intense staining, 2 (11.8%) showed medium staining, and 3 (17.6%) showed slight staining. No staining was described in 2 of 17 cases (11.8%) (Fig. 1).

### EOR vs. staining

In one case with GTR, no information about the staining grade was available. In the remaining 10 cases, 7 (70%) showed intense staining, one (10%) showed slight staining, and 2 (20%) showed no staining at all.

In those cases with STR (*n* = 3), one case showed intense, one case medium, and one without staining.

### Side effects

Except for yellow-colored urine, no side effects obviously related to fluorescein were found throughout the observation period. The most feared side effect – intraoperative hypotonia – could not be observed in any case of this study.

## Discussion

To safely enhance maximal EOR, different technical advancements have been introduced to neurosurgery, like an operating microscope, ultrasound [13], and neuronavigation [14]. Still, the surgeon must rely on visual and haptic clues during surgery, but with fluorescence-guided surgery – integrated with dedicated filters within the microscope – there is a beneficial tool for safely maximizing EOR in any patient suffering from almost any contrast-enhancing lesion at a meager cost and a low-risk profile at anyone’s disposal. This has been shown for a wide range of entities in adults, including but not limited to high-grade gliomas, metastases, hemangioblastomas, and gangliogliomas [5, 9, 10, 10, 12, 15, 16] and in children [17, 18].

In our hospital, we have a comprehensive experience with FL-guided surgery in neuro-oncology. After informed consent about FL and its off-label use, FL-guided surgery is performed on any patient not suffering from hepatic or renal comorbidities but suffering from a contrast-enhancing cerebral lesion.

In our experience from prior studies in adult neurosurgery, we have not seen any adverse effects related to the administration of FL when used for tumor surgery. This retrospective study aimed to assess the feasibility of FL-guided surgery in posterior fossa tumors in children and to outline the potential benefits of improved visualization. Following these findings in our adult patients, there was no adverse event related to the administration of FL in any of the included patients in this study. In contrast to other dyes, FL does not lead to a direct display of tumors but accumulates in the broken blood–brain barrier, similar to gadolinium [19].

The naked human eye can observe FL fluorescence at high doses [20]. The use, in combination with a specific filter built into the microscope, led to a significant dose reduction and a reduction of adverse events or complications [16].

In this study, FL was used to improve visualization and maximize the resection of posterior fossa tumors in children. The dosage of FL was reduced as compared to the dosage usually used for adults [15, 21]. This is a cautionary measure we used to reduce potential adverse events even further in children due to possible differences in metabolism.

Even with the reduced dose of FL, the staining was intense in most cases (58.8%). Besides temporary yellowish discoloration of the urine, we could not find severe adverse events in this study.

FL has been used widely and for a long time in ophthalmology. Thus, there is a considerable experience with its use in humans. Both in ophthalmology and neurosurgery, very few adverse events have been observed. The most common negative effect seems to be an anaphylactic reaction [22, 23].

To our knowledge, this is the first report evaluating low-dose FL in posterior fossa tumor surgery in exclusively pediatric patients. FL may substantially improve tumor visualization, resection, and patient safety in these cases. Typically, the grade of FL staining tumor tissue is proportional to the intensity of gadolinium enhancement preoperatively [19, 24, 25].

In our cohort, gadolinium enhancement was seen in every included case in the preoperative MRT but in variable intensities. As reported by the performing surgeon, the visibility of tumorous tissue was improved by FL and filter view and was considered helpful for complete tumor removal in most cases.

In the cases where a GTR was preoperatively considered possible and intended during the procedure, GTR was accomplished in 78.6%; 21.4% of these cases resulted in STR. In 2 of these 3 cases, the resection was stopped intentionally to preserve function or limit surgical morbidity.

In a similar study by Göker and Kırış [18] FL guidance with the YELLOW-560-nm filter was shown to be a safe and effective method in pediatric brain tumor surgery. The authors claimed that it would also be feasible to increase the extent of resection in this age group.

In our study, we achieved an EOR of 78,6% GTR in posterior fossa tumors in children when the tumor was preoperatively considered resectable. Compared to EOR rates in the literature concerning tumors in the posterior fossa in children, we did not see a significant difference. For instance, in a study by Bhatt et al., GTR complete resection rates for pilocytic astrocytomas, medulloblastomas, and ependymomas were 77%, 79%, and 63% without FL guidance [26]. In contrast to the aforementioned study, our study population is very heterogenous, and the patients were suffering from different entities of tumors in variable localizations (e.g., rhomboid fossa and pineal region), potentially leading to a covering of stained tissue for instance by overlaying eloquent brain tissue. Hopefully, in the future, these limitations could be overcome by endoscopes, including the FL filters.

Any fluorophore’s benefits during surgical treatment are based on adequate intraoperative exposure to visualize fluorescence. However, variable intraoperative circumstances may lead to incomplete resection, even in preoperatively considered completely resectable tumors, such as adjacent eloquent tissue or adherence to significant vessels. Further reasons could be a non-sufficient surgical window or hanging down of the overlying brain leading to inadequate visualization of tumor tissue.

The precise mechanism of action inducing the accumulation of FL in tumor tissue is still not known. There is a noticeable resemblance between the grade of FL staining, as seen intraoperatively, and the intensity of gadolinium enhancement in the preoperative MRI. Thus, the assumption that the impairment of the BBB is the principal mechanism in FL staining became a considerable theory and is regarded as established. At least for glioma cells, a breakdown of the BBB is assumed, enabling the permeation of FL into the extracellular space [6, 27, 28]. Neira et al. not only found intraoperative FL staining to be comparable to gadolinium uptake but also even discovered FL to have extended enhancing regions compared to gadolinium enhancement. This might be possible due to its lower molecular weight [19].

This study must be considered a preliminary or a feasibility study of the use of FL in posterior fossa surgery in children due to its limitations, for instance, regarding sample size and diversity in age and entity. Based on our current data, we cannot estimate the benefit concerning the overall extent of resection or outcome. Nevertheless, our personal experience was supported by our findings in the surgical reports, mentioning “helpful” or “intense” fluorescence in most of the cases and especially concerning the tumor margins particularly, and it was often observed to be helpful in distinguishing a minimal rest of tumor tissue from potentially eloquent brain tissue. Despite its low cost and despite having almost no drawbacks for the patient, there is still a lack of final approval of the drug in many countries by the respective competent authorities, irrespective of its potential benefits.

## Conclusions

FL is a widely and readily available method for fluorescence-guided tumor resection, enhancing the visualization of tumor margins intraoperatively resembling contrast enhancement in T1-weighted MRI. In combination with a specific filter, FL is a reliable, safe, and feasible tool in posterior fossa surgery in children. Further studies may hopefully elucidate issues like the outcome or extent of resection in a randomized, controlled clinical trial with adequate power to precisely assess them within a predefined observation period.

## Data Availability

Upon request, data and material can be provided by the corresponding author.

## Abbreviations

FL: Fluorescein sodium
MRI: Magnetic resonance imaging
MB: Medulloblastoma
ATRT: Atypical teratoid/rhabdoid tumor
PA: Pilocytic astrocytoma
BBB: Blood-brain barrier
EOR: Extent of resection
GTR: Gross total resection
OB: Open biopsy
STR: Subtotal resection
PPR: Planned partial resection
